# Inter-rater reliability of risk of bias tools for non-randomized studies

**DOI:** 10.1186/s13643-023-02389-w

**Published:** 2023-12-07

**Authors:** Isabel Kalaycioglu, Bastien Rioux, Joel Neves Briard, Ahmad Nehme, Lahoud Touma, Bénédicte Dansereau, Ariane Veilleux-Carpentier, Mark R. Keezer

**Affiliations:** 1 Faculty of Medicine, Université de Montréal, Montreal, QC Canada; 2 Department of Neurosciences, Université de Montréal, Montreal, QC Canada; 3 Centre Hospitalier de L’Université de Montréal, Pavillon R R04-700, 1000 Saint-Denis St., Montreal, QC H2X 0C1 Canada; 4 School of Public Health, Université de Montréal, Montreal, QC Canada

**Keywords:** ROB assessments, Systematic reviews, Neurology

## Abstract

**Purpose:**

There is limited knowledge on the reliability of risk of bias (ROB) tools for assessing internal validity in systematic reviews of exposure and frequency studies. We aimed to identify and then compare the inter-rater reliability (IRR) of six commonly used tools for frequency (Loney scale, Gyorkos checklist, American Academy of Neurology [AAN] tool) and exposure (Newcastle–Ottawa scale, SIGN50 checklist, AAN tool) studies.

**Methods:**

Six raters independently assessed the ROB of 30 frequency and 30 exposure studies using the three respective ROB tools. Articles were rated as low, intermediate, or high ROB. We calculated an intraclass correlation coefficient (ICC) for each tool and category of ROB tool. We compared the IRR between ROB tools and tool type by inspection of overlapping ICC 95% CIs and by comparing their coefficients after transformation to Fisher’s *Z* values. We assessed the criterion validity of the AAN ROB tools by calculating an ICC for each rater in comparison with the original ratings from the AAN.

**Results:**

All individual ROB tools had an IRR in the substantial range or higher (ICC point estimates between 0.61 and 0.80). The IRR was almost perfect (ICC point estimate > 0.80) for the AAN frequency tool and the SIGN50 checklist. All tools were comparable in IRR, except for the AAN frequency tool which had a significantly higher ICC than the Gyorkos checklist (*p* = 0.021) and trended towards a higher ICC when compared to the Loney scale (*p* = 0.085). When examined by category of ROB tool, scales, and checklists had a substantial IRR, whereas the AAN tools had an almost perfect IRR. For the criterion validity of the AAN ROB tools, the average agreement between our raters and the original AAN ratings was moderate.

**Conclusion:**

All tools had substantial IRRs except for the AAN frequency tool and the SIGN50 checklist, which both had an almost perfect IRR. The AAN ROB tools were the only category of ROB tools to demonstrate an almost perfect IRR. This category of ROB tools had fewer and simpler criteria. Overall, parsimonious tools with clear instructions, such as those from the AAN, may provide more reliable ROB assessments.

**Supplementary Information:**

The online version contains supplementary material available at 10.1186/s13643-023-02389-w.

## Introduction

Risk of bias (ROB) assessment is a critical step in a systematic review [1]. Accurate ROB assessments identify the degree of bias in different bodies of evidence to inform decisions made by health professionals and policy makers. Given that low-bias randomized controlled trials (RCTs) cannot always be conducted, many public health officials rely on observational studies to inform their medical policies [2]. Proper ROB assessment is especially important for these non-randomized observational studies, as various sources of bias (e.g., confounding bias) are more likely to arise than in their RCT counterparts [1]. Without reliable ROB tools, one may overestimate the validity of results from high-bias studies, which may lead to the incorrect synthesis of knowledge and incorrect guidance for policy makers [2].

The high number of ROB tools and the lack of guidance on their optimal use in non-randomized studies, particularly in descriptive or analytical observational studies, are major obstacles to the interpretation of systematic reviews. There is a growing number of domain- and design-specific ROB tools for non-randomized studies, especially for frequency and exposure studies in health-related systematic reviews. Frequency studies use cohort or cross-sectional designs to assess the incidence or prevalence of an outcome [3]. Through cohort and case–control studies, exposure study designs observe outcome occurrence in relation to a given exposure [4]. Several research organizations, such as the American Academy of Neurology (AAN), have created their own tools to evaluate these types of studies [5]. Other commonly used ROB tools for frequency studies include the Loney scale and the Gyorkos checklist, whereas for exposure studies the Newcastle–Ottawa scale and the SIGN50 checklist are highly used tools [6–9]. In general, for non-randomized interventional studies, Cochrane recommends the ROBINS-I tool to evaluate potential sources of bias. There are currently no practice standards for ROB tools in observational studies, possibly due to the limited knowledge on how these numerous tools compare to one another [10, 11].

These commonly used ROB tools have not previously reported inter-rater reliability, which attempts to quantify the performance of the tool by assessing the reproducibility of ratings between evaluators [1]. Furthermore, comprehensive head-to-head comparisons for these ROB tools are lacking [12]. There is a pressing need to identify and compare the inter-rater reliability of individual ROB tools to better guide their optimal use in systematic reviews of observational studies. As a primary objective, we aimed to quantify and then compare the inter-rater reliability of three commonly used ROB tools for frequency (Loney scale, Gyorkos checklist, AAN frequency tool) and for exposure (Newcastle–Ottawa scale, SIGN50 checklist, AAN exposure tool) studies. As secondary objectives, we identified and compared the inter-rater reliability of each category of ROB tool (scales, checklists, AAN tools) and evaluated the criterion validity of the AAN tools.

## Methods

We conducted a reliability study and reported our findings using the Guidelines for Reporting Reliability and Agreement Studies (GRRAS; Supplemental Material, Table S1) [13]. We defined frequency studies as descriptive studies that aimed to measure incidence or prevalence [3]. We defined exposure studies as analytical observational studies (e.g., cohort or case–control studies) that aimed to compare outcomes in two or more exposure groups [4]. These definitions are based on those generally used in the systematic review literature.

### Selection and description of the ROB tools

We first selected one AAN ROB tool designed for frequency studies and another for exposure studies. The AAN ROB assessment tools use a four-tier classification system, whereby each article is rated from class one (lowest ROB) to class four (highest ROB) [5]. Each rating has a distinct set of criteria tailored to the review question and study design. Although the AAN has various ROB tools, none was explicitly stated to be a frequency or exposure ROB tool. We therefore selected tools with the most fitting criteria for the intended type of study. For frequency studies, we chose the Population Screening Scheme, as this tool assessed characteristics needed for a high-quality frequency study, such as having a representative and unbiased sample population. For exposure studies, we chose the Prognostic Accuracy Scheme over the similar Causation Evidence Scheme as the latter had stricter criteria concerning confounding factors and biological plausibility. The precision of the criterion limited the tool’s scope and made it better suited to assess observational studies that were specifically implemented where randomized controlled trials could not be due to ethical concerns [5].

The two other categories of ROB tools considered in our study were scales and checklists (with or without summary judgments). Scales include a list of items that are each scored and assigned a weight. After scoring each weighted item, a quantitative summary score is produced [1]. For checklists, raters answer predetermined domain-specific questions from a given set of responses, such as “yes,” “no,” or “uncertain.” Although no instructions are provided to calculate an overall score, some checklists provide guidance to formulate a summary judgment, such as a low, intermediate, or high ROB [10].

We searched for two scales and two checklists from published systematic reviews which qualitatively described an extensive list of available ROB tools [1, 14, 15]. Over the period of June–August 2020, we searched for a combination of the following terms on Google Scholar: “Risk of Bias Tools,” “Observational Studies,” “Non-randomized studies,” “Exposure studies,” and “Frequency studies.” From this search, we found three systematic reviews, which each had a comprehensive list of various ROB tools, and five academic institutions that each created their own ROB tool [1, 9, 14–19]. We screened for a preliminary set of ROB tools for exposure and frequency studies from these systematic reviews and academic institutions by using the following criteria: (i) freely available online in English, (ii) simple to use for non-experts in ROB assessment, and (iii) commonly used for non-randomized studies of frequency or exposure. A ROB tool was considered simple to use for non-experts if there were no reviews stating it was “complicated” or “difficult to summarize” [1, 14, 15]. Two authors (IK and BR) then assessed the citation impact of each tool on PubMed and GoogleScholar to produce a list of five commonly used tools for each category of tool (scale, checklist) and for each study design (frequency, exposure; Supplemental Material, Table S2). Consensus for the final set of tools was settled through consensus with a third author (MRK) based on the initial set of criteria. We selected four ROB tools: the Loney scale and the Gyorkos checklist for frequency studies, as well as the Newcastle–Ottawa scale and the SIGN50 checklist for exposure studies (Table 1) [6–9]. Certain tools had various versions designed for specific study designs. We used the most appropriate version of these tools for each study design (frequency tools: case series/survey studies or cross-sectional designs; exposure tools: cohort or case–control designs). We followed the suggested summary scoring method for the Gyorkos and SIGN50 checklists [7, 9]. For the Loney and the Newcastle–Ottawa scales, we split the total score into 3 equal tiers (low, intermediate, and high ROB) to allow for category comparisons [6, 8].

**Table 1:** Risk of bias tools included

Tool (study design)	Tool content and judgment criteria	Reporting of results
**Frequency studies**		
Gyorkos et al. (cohort) [7]	Checklist, 4 categories • Selection of participants • Intervention/exposure • Outcome • Follow-up	Overall assessment into low risk of bias (few minor flaws and no major flaws), intermediate risk of bias (some minor flaws and no major flaws), high risk of bias (≥ 1 major flaw). No explicit guidance on the weight of individual items
Gyorkos et al. (cross-sectional) [7]	Checklist, 3 categories • Selection of participants • Intervention/exposure • Outcome	
Loney et al. (cohort & cross-sectional) [6]	Scale, 8 questions • Selection of participants • Statistical analyses • Validity of study methods • Applicability of results	Overall assessment into low risk of bias (6–8 points), intermediate risk of bias (3–5 points), high risk of bias (0–2 points). No explicit guidance on the weight of individual items
AAN ROB tool (population screening scheme) [5]	AAN tool, 4 classes • Selection of participants • Assessment of outcomes	Overall assessment into low risk of bias (class 1), intermediate risk of bias (class 2), high risk of bias (class 3) depending on criteria for each class. No weighting necessary. Class 4 articles (highest risk of bias) not considered in our study, as not used for published AAN guidelines
**Exposure studies**		
SIGN50 (cohort) [9]	Checklist, 14 questions • Research question • Selection of participants • Assessment of outcomes • Confounding • Statistical analyses	Overall assessment into low risk of bias (+ +), intermediate risk of bias ( +), high risk of bias ( −). No explicit guidance on the weight of individual items
SIGN50 (case–control) [9]	Checklist, 11 questions • Research question • Selection of participants • Assessment of outcomes • Confounding • Statistical analyses	
Newcastle–Ottawa scale (cohort) [8]	Scale, 8 questions • Selection of participants • Comparability of groups • Exposure	Each selected response may or may not be associated with a star. Overall assessment into low risk of bias (≥ 7 stars), intermediate risk of bias (4–6 stars), high risk of bias (≤ 3 stars). No explicit guidance on the weight of individual items
Newcastle–Ottawa scale (case–control) [8]	Scale, 8 questions • Selection of participants • Comparability of groups • Exposure	
AAN ROB (prognostic accuracy scheme) [5]	AAN scheme, 4 classes • Confounding • Assessment of outcomes	Overall assessment into low risk of bias (class 1), intermediate risk of bias (class 2), high risk of bias (class 3) depending on criteria for each class. No weighting necessary

### Article selection

We sampled 30 frequency and 30 exposure articles from randomly selected clinical practice guidelines of the AAN published between 2015 and 2020 (Supplemental Material, Tables S3 and S4). We selected articles from the AAN guidelines for convenience, as they were already assigned a ROB rating by the AAN. To ensure that we selected articles evaluated by the appropriate AAN ROB tool, we verified the appendices of these clinical guidelines which stated if the Population Screening Scheme (frequency studies) or the Prognostic Accuracy Scheme (exposure studies) were used to evaluate the included articles. The appendices outlined all articles by class; therefore, we used information from this section to choose an equal number of class one, class two, and class three ROB articles, as rated by the authors of the original AAN systematic reviews. Although the AAN has four classes of risk of bias, we only used articles from classes 1–3 for two reasons. Firstly, class four studies are not included in the AAN published guidelines given their high risk of bias; therefore, we could not choose any class four articles from the guidelines to be evaluated [5]. Secondly, to allow for comparisons between ROB tools, we needed to split ROB assessments into three levels, with class one articles as low ROB, class two articles as intermediate ROB, and class three articles as high ROB. Of note, although articles were selected from the AAN guidelines, the chosen studies included a diverse range of topics within neurology and medicine.

### Rating process

We recruited six raters (BR, JNB, AN, LT, BD, AVC), all of whom were post-graduate neurology residents at our institution who had previously completed at least one systematic review. All raters attended a 60-min course on the selected ROB tools to ensure a standardized familiarity with the instruments. During this course, the necessity of ROB tools in systematic reviews was discussed and a description of each tool along with their scoring system was given. After the training, participants were asked to rate articles independently (i.e., without communication between raters) using a customized online form. Each rater assessed all chosen 60 articles using a set of three tools for frequency (*n* = 30) and exposure (*n* = 30) studies. All the exposure and frequency tools were used by each rater on all the exposure and frequency studies, respectively. We varied the sequence of articles to be assessed across raters, as well as the order of ROB tools across both raters and articles. Raters were asked to limit themselves to a maximum of 10 articles per day to avoid exhaustion.

### Statistical analyses

We assessed inter-rater reliability with a two-way, agreement, average-measures intraclass correlation coefficient (ICC) with 95% confidence intervals (CI). This coefficient is commonly used to measure agreement on the ordinal scale for multiple raters [20]. We compared the inter-rater reliability between frequency tools (Loney, Gyorkos, and AAN frequency tool), exposure tools (Newcastle–Ottawa scale, SIGN50, and AAN exposure tool), and category of ROB tool (scales, checklists, and AAN tools) by transforming their ICC to Fisher’s *Z* values and testing the null hypothesis of equality. No adjustment for multiple testing was done. We also inspected their ICC and associated 95% CI. We visually inspected the variances across raters for each median score (for the pooled checklists, scales, and the AAN tools) and did not identify evidence of heteroscedastic variances. Homoscedasticity is a primary assumption behind the ICC, and violation of this assumption may inflate ICC estimates, which may lead to an overstatement of the inter-rater reliability [21]. Finally, we calculated an ICC for each of our six raters by comparing the ratings they produced with the AAN tools for each article to the ROB ratings published by the AAN for these same articles (criterion validity).

We expected an ICC for most tools of approximately 0.50 based on prior publications assessing the Newcastle–Ottawa scale [22]. We used Landis and Koch benchmarks to define inter-rater reliability as poor (ICC < 0), slight (0–0.20), fair (0.21–0.40), moderate (0.41–0.60), substantial (0.61–0.80), almost perfect (0.81–0.99), and perfect (1.00) [23]. To detect a statistical difference between an ICC of 0.20 (slight reliability) versus 0.50 with a group of 6 raters, a minimum of 27 studies was required assuming at least 80% power and an alpha of 0.05 [24]. This was our reason for choosing to include a priori 30 frequency (10 of each class) and 30 exposure studies (10 of each class), for a total of 60 articles. We used a threshold of *p* value < 0.05 for statistical significance and performed our analyses with R Studio (v.1.2.5) [25].

## Results

### Availability of data and materials

The datasets supporting the conclusions of this article are available at https://datadryad.org/stash/share/6PQuln5wyTvTBx_CO_JFESVD8M7gX1ImQAy4t4JVxls.

### Inter-rater reliability of ROB tools

**Fig. 1:**
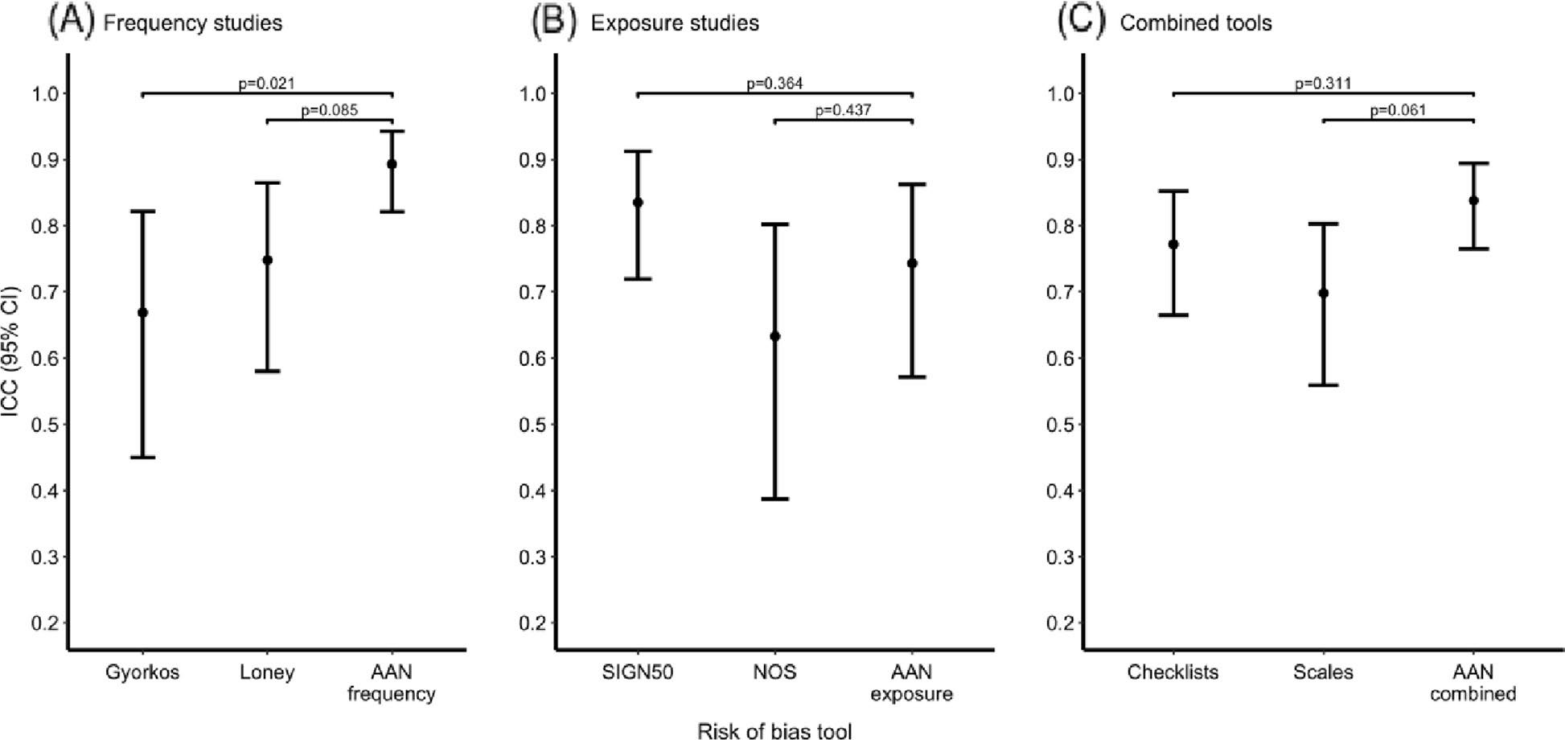
Intraclass correlation coefficient (ICC) by individual tools (**A**, **B**) and tool types (**C**). Abbreviations: AAN, American Academy of Neurology; CI, confidence interval; ICC, intraclass correlation coefficient; NOS, Newcastle–Ottawa scale

The SIGN50 (ICC = 0.835; 95% CI 0.719, 0.912) and the AAN frequency (ICC = 0.893; 95% CI 0.821, 0.943) tools had the highest ICC point estimates; these fell within the range of an almost perfect reliability (i.e., 0.81–0.99; Fig. 1, panels A and B). The four other tools had a substantial reliability (i.e., 0.61–0.80): the Loney scale (ICC = 0.749; 95% CI 0.580, 0.865), the Gyorkos checklist (ICC = 0.669; 95% CI 0.450, 0.821; Fig. 1A), the Newcastle–Ottawa scale (ICC = 0.633; 95% CI 0.387, 0.802), and the AAN exposure tool (ICC = 0.743; 95% CI 0.517, 0.862; Fig. 1B). The AAN frequency tool had higher inter-rater reliability than the Gyorkos checklist (*p* = 0.021). The AAN frequency tool trended to have a greater inter-rater reliability as compared to the Loney scale, with only minimal overlap in their 95% CIs (*p* = 0.085; Fig. 1A). We did not observe any other significant differences in ICC among the remaining tools. A summary of the results can be found in Supplemental Material, Table S5.

### Inter-rater reliability of categories of ROB tools

The AAN ROB tools, taken together as a category of ROB tool, had an almost perfect inter-rater reliability (ICC = 0.838; 95% CI 0.765, 0.894; Fig. 1C). The inter-rater reliability of scales (ICC = 0.698; 95% CI 0.559, 0.803) and checklists (ICC = 0.772; 95% CI 0.664, 0.852) were substantial. Although checklists did not differ significantly in inter-rater reliability when compared to the AAN ROB tools (*p* = 0.311), scales trended towards a lower inter-rater reliability compared to the AAN ROB tools (*p* = 0.061), with little overlap in their 95% CI. A summary of the results can be found in Supplemental Material, Table S6.

### Criterion validity of AAN ROB tools

We obtained the ICC using the AAN tools for each of our six reviewers as compared to the original ratings from the published AAN reviews. The average ICC among the six reviewers was moderate (0.563; 95% CI 0.239, 0.739). Individual point estimates for ICCs ranged from 0.417 (95% CI 0.022, 0.652) to 0.683 (95% CI 0.472, 0.810).

## Discussion

Several ROB tools are available to assess non-randomized studies; however, few have been thoroughly evaluated in terms of inter-rater reliability. Non-randomized studies, especially observational studies, usually harbor greater potential threats to their internal validity that deserve particular attention as compared to randomized studies. Reliable ROB tools for observational studies are therefore essential to properly appreciate and assess evidence from articles in systematic reviews.

In this inter-rater reliability assessment of ROB tools for exposure and frequency articles, we observed that all individual tools reached at least the substantial inter-rater reliability range (ICC point estimate = 0.61–0.80). We observed that the AAN tool for frequency studies had a higher inter-rater reliability as compared to the Gyorkos checklist and trended towards a higher inter-rater reliability as compared to the Loney scale. We did not observe differences in the inter-rater reliability for tools used in exposure studies (Newcastle–Ottawa scale, SIGN50 checklist, and AAN tool). When each category of ROB tool was analyzed, the AAN category of ROB tools was the only one to demonstrate an almost perfect inter-rater reliability, with trends in their favor as compared to ROB scales (Newcastle–Ottawa and Loney scales). These results suggest that the AAN ROB tools, especially the AAN frequency tool, may offer a high inter-rater reliability.

We observed a significantly higher inter-rater reliability for the AAN frequency tool when compared to the Gyorkos checklist. These results may be explained by differences in scoring structures between the Gyorkos checklist and the AAN frequency tool. The Gyorkos checklist was the only ROB instrument in our study to distinguish between minor and major flaws in ROB appraisal [7]. We suspect this stratification of the potential impact of biases added more complexity in the ratings and may have allowed for greater variation in responses between raters, particularly when compared to the parsimonious grading scheme of the AAN. Furthermore, the Gyorkos checklist was the only tool lacking instructions for each question [7]. Lack of guidance within the instrument may have led to varying interpretations of items. These results suggest that individual characteristics of ROB tools, such as their complexity and the lack of explicit guidance aimed at the raters may decrease their inter-rater reliability. In keeping with this, a way to enhance the Gyorkos checklist would be to simplify its scoring structure and add clearer instructions to guide its use.

The AAN category of ROB tools was the only category (i.e., as compared to scales and checklists) to show an almost perfect reliability. The simple criteria of the AAN tools may have contributed to their greater inter-rater reliability as these criteria are less susceptible to divergent interpretations. We did not, however, include any class 4 articles from the AAN ROB tools, which may have led to an overestimation of their inter-rater reliability. The AAN tools also trended towards a higher inter-rater reliability when compared to scales. Scales included in our study had a stricter grading scheme than the chosen AAN tools, which should theoretically have led to less variability amongst raters. An explanation for this may be that certain questions in our scales were much more open to interpretation than the relatively explicit AAN criteria. In addition, our scales comprised a greater set of criteria than the AAN ROB tools, which may have contributed to their higher inter-rater variability. Our checklists were just as complete as our scales, and yet, no difference was found between checklists and the AAN ROB tools. This may also be explained by the possibility that the questions in our scales were less objective than our checklists. Moving forward, a way to optimize scales would be to incorporate simpler, more straightforward criteria.

Our findings may be compared to previous studies on the Newcastle–Ottawa scale, as this is the only included tool that had already been assessed for inter-rater reliability [8, 22]. Oremus et al. assessed the inter-rater reliability of scales such as the Newcastle–Ottawa scale using novice student raters [22]. The inter-rater reliability in their study for the case–control and for the cohort version of the tool was fair (0.55, 95% CI − 0.18, 0.89) and poor (− 0.19, 95% CI − 0.67, 0.35), respectively. Here, we report an overall substantial reliability (0.633; 95% CI 0.387, 0.802) for both versions combined. Slight differences in reliability study designs might contribute to this small discrepancy. In the first study, raters all had different levels of experience and were new to quality ROB rating, whereas our raters were all neurology trainees with similar experience in systematic reviews and had participated in a 60-min training session [22].

The inter-rater reliability of the AAN tool type was almost perfect between our participants but varied between fair and substantial when compared to the ROB assessments from published AAN guidelines. Several sources of discrepancy may explain these results. First, the AAN ROB tools do not guide raters on how to respond when information needed for a criterion is not explicitly stated in the article. This is especially important if that specific criterion can change the class of the article. For example, many of the class one frequency articles were graded as class three by our raters. This often occurred when our raters felt that there were ambiguities in determining if the cohort under study came from a clinical center with or without a specialized interest in the outcome. Many raters could not find this information directly stated in certain class one articles, thereby assuming that the articles did not have this specific study cohort and would then rate these class one articles as class three articles. Although these articles met all the other criteria of a class one article, they were required to rate it as class three due to this criterion. Raters did not have the opportunity to consider if these ambiguities should impact the final ROB rating. It is possible that raters from the AAN leave room for interpretation of ambiguous information, especially when an article meets all other necessary criteria for a lower ROB level. Secondly, the moderate agreement of our raters as compared to the reference AAN ratings may be partly explained by a framing effect. It is possible that reviewers involved in AAN guidelines inexplicitly prioritized certain criteria when classifying ambiguous articles. In contrast, our raters all came from similar academic backgrounds, and it could be that they prioritized certain AAN criteria similarly to one another, but differently from other authors involved in AAN guidelines. As an example, some exposure studies rated as class one in the AAN guidelines were assigned as class two ROB by our raters, as many of them were retrospective studies. Class one and class two ROB categories in the AAN exposure tool share core criteria; however, class one studies require prospective data collection. Finally, certain criteria may be open to interpretation in the AAN tool. For example, a class three article requires a “narrow” spectrum of people with or without the disease, whereas a class one article requires a “broad” spectrum of people, yet these terms are not quantified. This lack of specification may explain why some of our raters assigned a class three ROB for articles considered as class one by AAN raters. Overall, in order to improve the AAN tools, it would be beneficial to add instructions addressing how to rate articles when information is presented ambiguously, particularly emphasizing if certain criteria should be prioritized in this case, as well as instructions to define all quantitative adjectives used in the criteria.

A high inter-rater reliability is necessary, but not sufficient, to reach a valid assessment of ROB. Other factors are also important to consider when choosing a tool to assess and report ROB in systematic reviews. The choice of ROB tool usually implies a tradeoff between completeness and complexity. More parsimonious tools such as those from the AAN may allow raters to assess relevant sources of bias faster than more complex tools while maintaining a high inter-rater reliability, as observed in our study. They may not, however, cover all potential sources of bias across different study settings and designs. Whether the focused scope of domains assessed in more parsimonious tools preserves the validity of ratings for more complex study designs remains unclear. Future studies assessing the validity of various tools, especially in other health-related domains, and how their content influences their validity and inter-rater reliability are needed to better understand how these tools compare to one another.

## Strengths and limitations

The strengths of our study include a comprehensive assessment of the reliability of a larger number of ROB tools and the inclusion of a larger number of raters as compared to prior publications [1, 11, 12, 15]. The ratings were independent and performed on a sizable sample of articles. Our study, however, has limitations. We included participants with a similar academic background and asked them to rate articles in their field of study, which may have inflated the inter-rater reliability as compared to what may be observed for a more heterogenous group of participants. We chose raters with a common medical background as we believed this was more likely to reflect the most frequent population of raters in systematic reviews of clinical data. Furthermore, although the selected articles were diverse in study topic, they were all chosen from the AAN guidelines. This enabled us to assess the criterion validity of the AAN ROB tools; however, it could have hindered the generalizability of our findings to other domains. The selected ROB tools do not have criteria relating solely to neurology studies, therefore selecting neurology articles from the AAN should not be a reason for these tools to perform better in this study than another study with articles from other medical domains. In the future, studies could address the above limitations in generalizability by incorporating a more heterogenous group of raters, with varying academic backgrounds and articles from varying medical domains. Another limitation to our study’s completeness is that we chose to assess inter-rater reliability as a first step to assess the reliability of these ROB tools; however, we did not assess intra-rater reliability. In addition, although we chose commonly used ROB tools, we did not select a wide range of ROB tools. In order for future studies to be more complete, both intra- and inter-rater reliability could be assessed within the same study, with a larger scale of ROB tools. Finally, we constructed a summary ROB score for each scale assessed in our study to allow for an ease of comparison between all tools. This could have influenced the results as the scales did not originally have a scoring system; the final ROB assessment was left up to the interpretation of the rater based on the answered questions. Future studies comparing the inter-rater reliability of scales with and without a strict scoring system would be necessary to assess the impact this modification had on our results.

## Conclusion

There is a growing body of available ROB tools for non-randomized studies, although information is generally lacking on their reliability. In this inter-rater reliability study, we assessed and compared six common ROB tools for frequency and exposure studies. We observed that the AAN category of ROB tools had an almost perfect reliability, while all other categories had a substantial inter-rater reliability. All exposure tools were comparable in reliability, yet amongst the frequency tools, the AAN frequency tool had a significantly higher inter-rater reliability as compared to the Gyorkos checklist and trended towards a higher inter-rater reliability when compared to the Loney scale. Our findings suggest that parsimonious ROB tools, such as those from the AAN, may contribute to a high inter-rater reliability. However, it remains uncertain how such minimal criteria affect the overall validity of ratings produced by these tools.

### Supplementary Information

**Additional file 1:** **Table S1.** GRRAS Checklist. **Table S2.** Preliminary ROB Tool List. **Table S3.** Information for frequency articles. **Table S4.** Information for exposure articles. **Table S5.** Inter-rater reliability per ROB tool. **Table S6.** Inter-rater reliability per ROB tool category

## Data Availability

The datasets generated and analyzed during the current study are available in the dryad repository, https://datadryad.org/stash/share/6PQuln5wyTvTBx_CO_JFESVD8M7gX1ImQAy4t4JVxls

## Abbreviations

ROB: Risk of bias
AAN: American Academy of Neurology
ICC: Intraclass correlation coefficient
CI: Confidence intervals
