# Supplementary material for: Inter-rater reliability of risk of bias tools for non-randomized studies
Source: Syst Rev. 2023 Dec 7;12:227. doi: 10.1186/s13643-023-02389-w (PMC10702000; doi:10.1186/s13643-023-02389-w)
Supplement: Supplementary file 1 — Additional file 1: Table S1. GRRAS Checklist. Table S2. Preliminary ROB Tool List. Table S3. Information for frequency articles. Table S4. Information for exposure articles. Table S5. Inter-rater reliability per ROB tool. Table S6. Inter-rater reliability per ROB tool category [file 13643_2023_2389_MOESM1_ESM.pdf]

SUPPLEMENTAL MATERIAL

Title

Inter-rater Reliability of Risk of Bias Tools for Non-Randomized Studies

Authors and affiliations

Kalaycioglu, Isabel[1]; Rioux, Bastien[2,3]; Neves Briard, Joel[2,3]; Nehme, Ahmad[2,3]; Touma, Lahoud[2,3]; Dansereau, Bénédicte[2,3]; Veilleux-Carpentier, Ariane[2,3]; Keezer, Mark R[2,3,4]

[1] Faculty of Medicine, University of Montreal, Montreal, Quebec, Canada

[2] Department of Neurosciences, University of Montreal, Montreal, Quebec, Canada

[3] Centre hospitalier de l'Université de Montréal, Montreal, Quebec, Canada

[4] School of Public Health, University of Montreal, Montreal, Quebec Canada

Corresponding author

Mark R Keezer

Centre Hospitalier de l'Université de Montréal, Pavillon R R04-700

1000 Saint-Denis St., Montreal, QC, Canada, H2X 0C1

Telephone: +01 514 890 8233, fax: +01 514 412 7139

Email: mark.keezer@umontreal.ca

Table of contents

Table S1. GRRAS Checklist. 3
Table S2. Preliminary ROB Tool List. 4
Table S3. Information for frequency articles. 6
Table S4. Information for exposure articles. 11
Table S5. Inter-rater reliability per ROB tool. 16
Table S6. Inter-rater reliability per ROB tool category. 16
References 17

Table S1. GRRAS Checklist.

Section	Item #	Checklist item	Reported on page #
Title/Abstract	1	Identify in title or abstract that interrater/intrarater reliability or agreement was investigated.	3-4
Introduction	2	Name and describe the diagnostic or measurement device of interest explicitly.	5
	3	Specify the subject population of interest.	5
	4	Specify the rater population of interest (if applicable).	5
	5	Describe what is already known about reliability and agreement and provide a rationale for the study (if applicable).	5-6
Methods	6	Explain how the sample size was chosen. State the determined number of raters, subjects/objects, and replicate observations.	8-9
	7	Describe the sampling method.	7-8
	8	Describe the measurement/rating process (e.g. time interval between repeated measurements, availability of clinical information, blinding).	8
	9	State whether measurements/ratings were conducted independently.	8
	10	Describe the statistical analysis.	8-9
Results	11	State the actual number of raters and subjects/objects which were included and the number of replicate observations which were conducted.	10
	12	Describe the sample characteristics of raters and subjects (e.g. training, experience).	8
	13	Report estimates of reliability and agreement including measures of statistical uncertainty.	10
Discussion	14	Discuss the practical relevance of results.	11-15
Auxiliary material	15	Provide detailed results if possible (e.g. online).	Supplemental material

Table S2. Preliminary ROB Tool List.

**Frequency ROB Tools**	
**Scales**	**Citation Impact (#)**
MINORS(1)	Pubmed: 710<br>Googlescholar: 2289
Loney 1998(2)	Pubmed: 123<br>GoogleScholar: 488
Fowkes(3)	Pubmed: 73<br>Googlescholar: 358
DuRant(4)	Pubmed: 12<br>Googlescholar: 57
Silva(5)	Pubmed: 5<br>Googlescholar: 46
**Checklists**	**Citation Impact (#)**
Gyorkos(6)	Pubmed: 13<br>Googlescholar: 115
Munn et al(7)	Pubmed: 117<br>Googlescholar: 365
Joanna Briggs Institute(8)	Pubedmed: No results<br>Googlescholar: 180
Shamliyan(9)	Pubmed: 48<br>Googlescholar: 162
CASP(10)	Pubmed: searched for: critical appraisal skills programme checklist (cited by 5) , searched for: critical appraisal skills programme (cited by 15)<br>Googlescholar: 149
**Exposure ROB Tools**	
**Scales**	**Citation Impact (#)**
Newcastle Ottawa Scale(11)	Pubmed: 2,708<br>Googlescholar: 6093
MINORS(1)	Pubmed: 710<br>Googlescholar: 2289
EPA-IRIS(12)	Pubmed: searched for: EPA IRIS (cited by 0)<br>Googlescholar: searched for: EPA IRIS, review of EPAs integrated risk information

	system (IRIS) process (cited by 85), searched for: Integrated risk information system (IRIS) (cited by 147)
Genaidy(13)	Pubmed: 24<br>Googlescholar: 108
OHAT(14)	Pubmed: searched for: OHAT Risk of Bias Rating Tool (cited by 6) Googlescholar: 3

**Checklists**	**Citation Impact (#)**
SIGN50(15)	Pubmed: 13<br>Googlescholar: 194
Joanna Briggs Institute(8)	Pubmed: No results<br>Googlescholar: 180
Shamliyan(9)	Pubmed: 48<br>Googlescholar: 162
CASP(10)	Pubmed: searched for: critical appraisal skills programme checklist (cited by 5) , searched for: critical appraisal skills programme (cited by 15)<br>Googlescholar: 14
Zaza et al(16)	Pubmed: 5<br>Googlescholar: 59

Table S3. Information for frequency articles.

Class 1 risk of bias (lowest risk of bias)

Author and Year	Study Objective	Study Design and Population
**Ackers, 2011**	To identify cases and causes of death in a pediatric patient cohort prescribed anti epileptic drugs (AEDs).	Retrospective cohort study of 6,190 participants between the ages of 0-18 years, who were seen by primary care services in the United Kingdom, registered in the General Practice Research Database between 1993 and 2005.
**Granbichler, 2015**	To calculate standardized mortality ratios (SMRs), identify epilepsy-related deaths, and estimate sudden unexpected death in epilepsy (SUDEP) rates in patients treated with vagus nerve stimulation (VNS) for epilepsy.	Retrospective cohort study of 466 patients who received VNS between January 1, 1995 and December 31, 2010 at King's College Hospital, London, United Kingdom.
**Langan, 1998**	To establish the incidence of sudden unexpected death in epilepsy (SUDEP) in the population of South Dublin and Wicklow with epilepsy.	Retrospective cohort study of participants with epilepsy in South Dublin and Wicklow between May 1992 to 1994.
**Derby, 1996**	To estimate the incidence of sudden unexpected death in epilepsy (SUDEP) among subjects with refractory epilepsy.	Cohort of refractory epilepsy patients on two or more anticonvulsants between the period of July 1, 1989, through November 30, 1992 in the United Kingdom.
**Lopez, 2007**	To examine the incidence of dementia in subjects with mild cognitive impairment (MCI).	Prospective cohort of 136 subjects from the Cardiovascular Health Study Cognition Study of Pittsburgh, classified as having MCI in 1998 and 1999.
**Fei, 2009**	To determine the prevalence of cognitive impairment, no dementia (CIND) among the elderly and to investigate potential predictive factors for its occurrence.	Cross sectional study, conducted in March of 2006, with community-dwelling residents aged over 65 years, in Taiyuan, China.
**Lopez, 2003**	To examine the prevalence of mild cognitive impairment (MCI) and its diagnostic classification in the cardiovascular health study (CHS) cognition study.	Cross sectional, multicenter study of 3,608 participants from the CHS, who had an MRI scan between 1991-1994.

**Hanninen, 2002**	To determine the prevalence of mild cognitive impairment (MCI) in an elderly population.	Cross sectional study of 806 subjects between the ages of 60-76, from the Kuopio population in eastern Finland.
**Messina, 2010**	To establish the prevalence of congenital muscular dystrophies (CMD) and cognitive impairment, the frequency of individual genetically defined forms and the presence of distinct phenotypes not associated with mutations in the known gene in the CMD population.	Cross sectional study with 92 participants selected from the patients followed in all of the Italian tertiary neuromuscular centers for CMD.
**Ganguli, 2004**	To estimate the prevalence and examine the course of mild cognitive impairment (MCI), amnestic type, using current criteria, within a representative community sample.	Cross sectional study with retroactive application of MCI criteria to a sample of 1,248 subjects drawn from voter registration lists in the rural mid-Monongahela Valley of southwestern Pennsylvania.

Class 2 risk of bias (intermediate risk of bias)

Author and Year	Study Objective	Study Design and Population
**Kivipelto, 2001**	To evaluate the impact of midlife elevated serum cholesterol levels and blood pressure on the subsequent development of mild cognitive impairment (MCI) and to investigate the prevalence of MCI in the elderly.	Cross sectional study of 1,449 subjects selected from the North Karelia Project and the FINMONICA (Finnish Multinational Monitoring of Trends and Determinants in Cardiovascular Disease) study during 1972, 1977, 1982, and 1987.
**Tennis, 1995**	To measure the incidence of sudden unexplained death in treated persons with epilepsy (SUDEP) and to identify risk factors for SUDEP.	Retrospective study of a cohort of 3,688 subjects between the ages of 15-49, with more than four prescriptions for anti epileptic drugs (AEDs), identified from the Saskatchewan health prescription drug file.
**Skandsen, 2008**	To assess the long-term outcome in survivors after severe head injury and relate outcome to injury severity.	Retrospective study of a cohort of 146 individuals admitted to the University Hospital of Trondheim, for severe head injuries during 1998-2002.
**Walczak, 2001**	To determine the incidence of and risk factors for sudden unexpected death in epilepsy.	Prospective cohort study of 4,578 patients from three epilepsy centers (MINCEP Epilepsy Care, Minneapolis,

		MN; Mayo Clinic Epilepsy Divi- sion, Rochester, MN; and Marshfield Clinic Epilepsy Section, Marshfield, WI) from June 1, 1991 to December 31, 1996.
**Wohlgemuth, 2004**	To investigate the prevalence of, and risk factors for, respiratory insufficiency in facioscapulohumeral muscular dystrophy (FSHD).	Cross sectional study with 10 eligible FSHD patients registered from four Dutch Centers on home ventilatory support between May 1999 and February 2000.
**Lee, 2009**	To investigate the influence of varying applications of diagnostic criteria on the prevalence of mild cognitive impairment (MCI).	Cross sectional study of randomly sampled group of 1,118 Korean elders aged 65 years or older living in Seongnam, Korea, on August 1, 2005.
**Hilal, 2013**	To study the prevalence of and associated factors for cognitive impairment and dementia in community dwelling persons from Singapore.	Cross sectional study with 4,605 community dwelling persons from Singapore, aged 40–85 years, who participated in the Singapore Chinese Eye Study (SCES).
**Gavrila, 2009**	To estimate the prevalence of amnestic mild cognitive impairment (aMCI), cognitive impairment, no dementia (CIND) and dementia in a general elderly population and to examine the associated socio-demographic factors.	Cross sectional study, where 1,074 individuals, aged 65-96 years, were sampled from the Murcia Region of southeastern Spain.
**Ding, 2015**	To investigate the prevalence of mild cognitive impairment (MCI) in the Chinese population.	Cross sectional study that evaluated 2,985 Chinese individuals without dementia, above the age of 60 years, residing in Shanghai.
**Luauté, 2010**	To compare the long-term functional outcome, improvement or deterioration, of patients considered in a vegetative state (VS) or a minimally conscious state (MCS) one year after coma onset, then yearly for up to 5 years.	Retrospective cohort study of 12 patients in VS and 39 in MCS between 1997 and 2004, selected from various intensive care units across France.

Class 3 risk of bias (highest risk of bias)

Author and Year	Study Objective	Study Design and Population
**Ruggles, 2001**	To assess the incidence, etiology, anti epileptic drug (AED) use and quality of life in patients having their first seizure at age 50 years or older.	Prospective cohort study with patients from the Marshfield Epidemiological Study Area (MESA), aged 50 years or older, who had their first seizure between July 1, 1996 and June 30, 1998.
**Pane, 2012**	To assess respiratory and cardiac function in a large cohort of patients with congenital muscular dystrophies (CMD) with reduced glycosylation of alphadystroglycan (a-DG).	Retrospective cohort study of 115 patients with a diagnosis of CMD with a-DG deficiency, recruited from two previous large multi-centric studies report- ing muscle, eye and brain involvement and motor abilities.
**Trevisan, 2006**	To determine the frequency of symptomatic or asymptomatic heart abnor- malities in a series of patients affected by facioscapulohumeral muscular dystrophy (FSHD).	Cross sectional study of a sample of 83 patients with FSHD, referred to three italian neuromuscular centers, located at the universities of Padua, Turin and Verona from 1998 onwards.
**Pascarella, 2016**	To estimate the occurrence of remote epileptic seizures and epileptiform activity in vegetative state (VS) and minimally conscious state (MCS) patients.	Prospective cohort study of patients with prolonged disorder of consciousness (DOC) due to traumatic, vascular or anoxic, etiology admitted to a neurorehabilitation unit for Disorders of Consciousness at Salvatore Maugeri Foundation (Telese Terme, Italy), from January 2005 to December 2012.
**Clement, 2012**	To assess the relative frequency of congenital muscular dystrophy (CMD) subtypes within the patient population referred to the The Dubowitz Neuromuscular Centre.	Cross sectional retrospective study of 214 UK patients referred to The Dubowitz Neuromuscular Centre. for assessment of 'possible CMD' between 2001 and 2008.
**Peat, 2008**	To determine the frequency of all known forms of congenital muscular dystrophy (CMD) in a large Australasian cohort.	Cross sectional retrospective study of patients referred to the University of Sydney between 1979 to 1996, combined with a prospective study of patients referred to The Children's Hospital in Westmead, until June 2006.

**Bagnato, 2017**	To evaluate the first clinical signs denoting emergence from unresponsive wakefulness syndrome (UWS).	Prospective cohort study of 31 patients with UWS, admitted to the Unit for Severe Acquired Brain Injuries for intensive rehabilitation after an acute brain injury in Italy.
**Statland, 2013**	To investigate the frequency of Coats syndrome and its association with D4Z4 contraction size in patients with facioscapulohumeral muscular dystrophy type 1 (FSHD1).	Cross sectional study of 5,357 patients with genetically confirmed FSHD (D4Z4 fragment) from the National Registry of FSHD Patients and Family Members, and 551 patients from the University of Rochester (UR)-FSHD database.
**Hui, 2001**	To describe the relapse rate after a first convulsion and factors associated with recurrence of seizure.	Retrospective cohort study, of 2,938 patients referred to the EEG lab from June 1993 to December 1996 at the Prince of Wale Hospital in Hong Kong.
**Guy-Coichard, 2008**	To study the characteristics of pain in patients suffering from neuromuscular disorders (NMD), in a large patient sample.	Cross sectional study including 862 patients from 30 french adult consult centers for NMD.

Table S4. Information for exposure articles.

Class 1 risk of bias (lowest risk of bias)

Author and Year	Study Objective	Study Design and Population
**Wikkelso, 2013**	To determine the sensitivity, specificity, positive and negative predictive values of the CSF Tap Test and resistance to CSF outflow for shunting outcome.	Prospective cohort, multi-center study with 115 patients with idiopathic normal pressure hydrocephalus, across nine European countries.
**Eilander, 2005**	Identify prognostic variables (e.g. Level of consciousness (LOC) at admission, time interval between brain injury and admission) to the LOC at discharge for patients with brain injury.	Retrospective cohort study where 145 patients, under the age of 25, were admitted to the Early Intensive Neurorehabilitation Programme for a prolonged unconscious state after severe brain injury, in the Netherlands.
**O'Loughlin, 1993**	To determine the frequency of and risk factors for falls and injurious falls in the noninstitutionalized elderly.	Prospective cohort study including 409 community-dwelling persons aged 65 years or more in west-central Montreal, Quebec, Canada.
**Heindl, 1996**	To describe the differences in outcome after persistent vegetative state (PVS), following traumatic or hypoxic brain injury and to find possible prognostic factors.	Prospective cohort, single center study with 127 patients with hypoxic or traumatic brain injury in Germany.
**Tinetti, 1988**	To identify the risk factors of falling in the elderly.	Prospective cohort study including 336 community-dwelling persons above the age 75 from the Yale Health and Aging Project.
**Hauser, 1990**	To identify seizure recurrence within a four year follow up period after the first unprovoked seizure.	Prospective cohort study, including 208 patients from four hospitals affiliated with University of Minnesota.
**Estraneo, 2013**	To identify prognostic markers to help predict recovery of responsiveness in postanoxic vegetative state patients.	Prospective cohort study of 43 inpatients with prolonged anoxic vegetative state, admitted to Unit for Disorders of Consciousness at the Institute for Rehabilitation, Salvatore Maugeri Foundation, Italy.
**Campbell, 1989**	To identify factors associated with falls in elderly subjects.	Prospective cohort community-based study, with

		761 subjects, aged 70 or older, in Mosgeil, New Zealand.
**Boyle, 2006**	To examine the extent to which persons with mild cognitive impairment (MCI) have an increased risk of Alzheimer disease (AD) and a more rapid rate of decline in cognitive function compared to similar persons without cognitive impairment.	Prospective cohort community-based study, with 786 subjects from the Rush Memory and Aging Project, Chicago.
**Scheller, 2015**	To investigate if quadrivalent HPV (qHPV) vaccination is associated with an increased risk of multiple sclerosis and other demyelinating sclerosis.	Retrospective cohort study, of 3,983,824 females between the ages of 10 to 44 years, in Denmark and Sweden, from 2006 to 2013.

<u>Class 2 risk of bias (intermediate risk of bias)</u>

Author and Year	Study Objective	Study Design and Population
**Langer-Gould, 2014**	To determine whether vaccines, particularly those for hepatitis B and Human papilloma virus, increase the risk of multiple sclerosis or other central nervous system demyelinating syndromes.	Nested case control study, with 780 patients both with and without demyelinating syndromes, selected from members of the Kaiser Permanente Southern California (KPSC) group, in California.
**Hesdorffer, 2011**	To pool data from four published case control studies of sudden unexpected death in epilepsy (SUDEP), to increase the power to determine risk factors.	Combined case control study from four separate studies, done by the U.S. (Walczak et al., 2001), Sweden (Nilsson et al., 1999), Scotland (Hitiris et al., 2007), and England (Langan et al., 2005), with a final sample of 289 SUDEP cases and 958 epilepsy without SUDEP controls.
**Hitiris, 2007**	To investigate the association between clinical characteristics and sudden unexpected death in epilepsy (SUDEP), focusing on likely risk factors.	Case control study with 6,140 patients registered with the Epilepsy Unit at the Western Infirmary in Glasgow, Scotland between 1982 and 2005.
**Hopkins 1988**	To identify the recurrence of seizure after the first unprovoked seizure and it's prognostic factors.	A prospective cohort study following 408 adults who were referred by a neurologist or physician after their first seizure to St Bartholomew's Hospital, London.

**Surges, 2010**	To determine whether abnormal cardiac repolarization and other electrocardiography (ECG) predictors for cardiac mortality occur in epilepsy patients and whether they are associated with an increased risk for sudden unexpected death in epilepsy (SUDEP).	Matched pair case control study, with 38 patients from the National Hospital for Neurology and Neurosurgery in London, United Kingdom.
**Lhatoo, 2010**	The objective was to determine the association between postictal generalized electroencephalographic (EEG) suppression (PGES), as a possible identifiable EEG marker of profound postictal cerebral dysfunction, and sudden unexpected death in epilepsy.	Matched case control study with 10 adult cases and 30 live controls selected from the department of neurophysiology, Frenchay Hospital, Bristol, United Kingdom between January 1997 and January 2009.
**Annegers, 1986**	To determine the patterns of risk for recurrence after an initial unprovoked seizure.	Population based retrospective cohort study from Rochester, Minnesota, U.S.A, following 424 participants after their first seizure during the period 1935-1979.
**Surges, 2011**	To examine whether the occurrence of postictal generalized electroencephalographic suppression (PGES) depends on seizure type and whether PGES occurs more frequently in people with epilepsy who died suddenly.	Case control study of 57 participants with focal epilepsies who underwent presurgical assessment from the National Hospital for Neurology and Neurosurgery, London, United Kingdom from 1992-2007.
**Nilsson, 1999**	To investigate the association between some clinical variables and sudden unexpected death in epilepsy (SUDEP) to identify risk factors.	Nested case control study based on participants between the ages of 15 and 70 years, who had been admitted to any hospital in Stockholm with the diagnosis of epilepsy during 1980–1989.
**Aurlien, 2012**	To estimate the incidence of sudden unexpected death in epilepsy (SUDEP) and to investigate whether use of lamotrigine (LTG) was associated with increased risk in female patients or other subgroups.	Case control study, with 26 SUDEP cases identified from the Norwegian Cause of Death Registry and matched controls identified from the Stavanger University Hospital, with a diagnosis of epilepsy between the period August 1, 1995–July 31, 2005.

**Class 3 risk of bias (highest risk of bias)**		
**Author and Year**	**Study Objective**	**Study Design and Population**
**Bagnato, 2012**	To determine whether the BDNFMet polymorphism plays a role in the recovery of consciousness and cognitive functions in patients in a vegetative state (VS) after traumatic brain injury (TBI).	Prospective cohort study of 53 patients in a VS, one month after a TBI, from five Italian centers specializing in the rehabilitation of post- traumatic disorders of consciousness.
**Gasperini, 1995**	To identify risk factors for acute exacerbations in multiple sclerosis.	Matched case control study of 89 patients with relapsing and remitting multiple sclerosis between January to December 1992, from the multiple sclerosis center of the University of Rome.
**Zheng, 2013**	To investigate if somatosensory evoked potential can accurately predict outcome in prolonged coma patients with diffuse axonal injury.	Prospective cohort study including 43 prolonged coma patients with diffuse axonal injury, admitted to the First Affiliated Hospital, School of Medicine, Zhejiang University in China.
**Lovell, 2003**	To evaluate memory dysfunction and self-reporting of symptoms in a group of athletes with concussion.	Prospective cohort study, with 64 high school athletes who had suffered concussion, recruited from a single high school in Pennsylvania.
**Collins, 2003**	To investigate whether postconcussion headaches are associated with neurocognitive and other symptom impairment.	Prospective cohort study of 109 participants selected from 20 high schools enrolled in the University of Pittsburgh Medical Center Sports Concussion Program within the states of Pennsylvania, Michigan, Illinois, Oregon, and Maine.
**Collins, 2002**	To investigate the relationship between concussion history in high school athletes and the on-field presentation of symptoms after subsequent concussion.	Prospective cohort study of 60 athletes without concussion history and 28 athletes with concussion history from the Sports Medicine Concussion Program offered by the University of Pittsburgh Medical Center.
**Chen, 2008**	To examine functional brain activation patterns before and after postconcussive symptoms (PCS) resolution.	Prospective cohort study of nine symptomatic concussed athletes compared to six healthy athletes

		referred to the McGill Sports Medicine Clinic.
**Patel, 2012**	To investigate the relationship between the phosphorylated tau/amyloid beta 1-42 (ptau/Ab1-42) ratio in ventricular cerebrospinal fluid and shunt outcome in patients with idiopathic normal pressure hydrocephalus (iNPH).	Prospective cohort study of 39 patients with suspected iNPH referred to a hospital affiliated with University of Pennsylvania.
**Iverson, 2006**	To examine whether athletes with a history of one or two previous concussions differed in their preseason neuropsychological test performances or symptom reporting.	Prospective cohort study of 867 male high school and university amateur athletes who completed preseason testing with Impact version 2.0.
**Iverson, 2004**	To examine the possibility that athletes with multiple concussions show cumulative effects of injury.	Prospective cohort study of 38 amateur athletes from the University of Pittsburgh Medical Center Sports Concussion Program.

Table S5. Inter-rater reliability per ROB tool.

ROB Tool	ICC (95% CI)	p-value
**Frequency studies**		
Loney Scale	0.580, 0.865	0.749
Gyorkos Checklist	0.450, 0.821	0.669
AAN Frequency Tool	0.821, 0.943	0.893
**Exposure studies**		
Newcastle-Ottawa Scale	0.387, 0.802	0.633
SIGN50 Checklist	0.719, 0.912	0.835
AAN Exposure Tool	0.517, 0.862	0.743

Abbreviations: CI, confidence interval; ICC, intraclass correlation coefficient.

Table S6. Inter-rater reliability per ROB tool category.

ROB Tool Category	ICC (95% CI)	p-value
Scales	0.559, 0.803	0.698
Checklists	0.664, 0.852	0.772
AAN ROB Tools	0.765, 0.894	0.838

Abbreviations: CI, confidence interval; ICC, intraclass correlation coefficient.

References

1. Slim, K., et al., Methodological index for non-randomized studies (minors): development and validation of a new instrument. ANZ J Surg, 2003. 73(9): p. 712-6.
2. Loney PL, Chambers LW, Bennett KJ, Roberts JG, Stratford PW. Critical appraisal of the health research literature: prevalence or incidence of a health problem. Chronic Dis Can. 1998;19(4):170-6.
3. Fowkes, F.G. and P.M. Fulton, Critical appraisal of published research: introductory guidelines. BMJ (Clinical research ed.), 1991. 302(6785): p. 1136-1140.
4. DuRant, R.H., Checklist for the evaluation of research articles. J Adolesc Health, 1994. 15(1): p. 4-8.
5. Luis Carlos Silva, P.O., María Paz Rodríguez, Sylvia Robles, A tool for assessing the usefulness of prevalence studies done for surveillance purposes: the example of hypertension. 2001.
6. Gyorkos TW, Tannenbaum TN, Abrahamowicz M, Oxman AD, Scott EA, Millson ME, et al. An approach to the development of practice guidelines for community health interventions. Can J Public Health. 1994;85 Suppl 1:S8-13.
7. Munn, Z., et al., The development of a critical appraisal tool for use in systematic reviews addressing questions of prevalence. Int J Health Policy Manag, 2014. 3(3): p. 123-8.
8. Moola S, M.Z., Tufanaru C, Aromataris E, Sears K, Sfetcu R, Currie M, Qureshi R, Mattis P, Lisy K, Mu P-F., Chapter 7: Systematic reviews of etiology and risk in Joanna Briggs Institute Reviewer's Manual, M.Z. Aromataris E, Editor. 2017, The Joanna Briggs Institute: The Joanna Briggs Institute.
9. Shamliyan, T., R.L. Kane, and S. Dickinson, A systematic review of tools used to assess the quality of observational studies that examine incidence or prevalence and risk factors for diseases. J Clin Epidemiol, 2010. 63(10): p. 1061-70.
10. National Collaborating Centre for Methods and Tools. in Webinar Companion : Spotlight on KT Methods and Tools. Episode 3. Hamilton, ON: McMaster University.
11. Stang, A., Critical evaluation of the Newcastle-Ottawa scale for the assessment of the quality of nonrandomized studies in meta-analyses. Eur J Epidemiol, 2010. 25(9): p. 603-5.
12. ACA Submits Comments to EPA Supporting Scientific Evaluation of Formaldehyde as Part of Draft IRIS. Jct Coatingstech, 2010. 7(11): p. 20-20.
13. Genaidy, A.M., et al., *An epidemiological appraisal instrument - a tool for evaluation of epidemiological studies.* Ergonomics, 2007. **50**(6): p. 920-60.
14. OHAT, *OHAT Risk of Bias Rating Tool for Human and Animal Studies.* 2015.
15. (SIGN), S.I.G.N., *A guideline developer's handbook.* 2019, SIGN: Edinburgh.
16. Zaza, S., et al., *Methods for conducting systematic reviews of the evidence of effectiveness and economic efficiency of interventions to reduce injuries to motor vehicle occupants.* Am J Prev Med, 2001. **21**(4 Suppl): p. 23-30.
